# Assessment of Autologous Blood marker localIzation and intraoperative coLonoscopy localIzation in laparoscopic colorecTal cancer surgery (ABILITY): a randomized controlled trial

**DOI:** 10.1186/s12885-023-10669-w

**Published:** 2023-03-03

**Authors:** Ke-hui Zhang, Jing-ze Li, Hai-bin Zhang, Ren-hao Hu, Xi-mao Cui, Tao Du, Liang Zheng, Shun Zhang, Chun Song, Mei-dong Xu, Xiao-hua Jiang

**Affiliations:** 1 Department of Gastrointestinal Surgery, Shanghai East Hospital, Tongji University, Shanghai, P. R. China 200120; 2 Center of Digestive Endoscopy, Shanghai East Hospital, Tongji University, Shanghai, China; 3 Research Center for Translational Medicine, Shanghai East Hospital, Tongji University, Shanghai, China

**Keywords:** Colorectal cancer, Laparoscopic surgery, Tumor localization, Endoscopic tattooing, Autologous blood, Intraoperative colonoscopy

## Abstract

**Background:**

Laparoscopic colorectal surgery has been proved to have similar oncological outcomes with open surgery. Due to the lack of tactile perception, surgeons may have misjudgments in laparoscopic colorectal surgery. Therefore, the accurate localization of a tumor before surgery is important, especially in the early stages of cancer. Autologous blood was thought a feasible and safe tattooing agent for preoperative endoscopic localization but its benefits remain controversial. We therefore proposed this randomized trial to the accuracy and safety of autogenous blood localization in small, serosa-negative lesion which will be resected by laparoscopic colectomy.

**Methods:**

The current study is a single-center, open-label, non-inferiority, randomized controlled trial. Eligible participants would be aged 18–80 years and diagnosed with large lateral spreading tumors that could not be treated endoscopically, malignant polyps treated endoscopically that required additional colorectal resection, and serosa-negative malignant colorectal tumors (≤ cT3). A total of 220 patients would be randomly assigned (1:1) to autologous blood group or intraoperative colonoscopy group. The primary outcome is the localization accuracy. The secondary endpoint is adverse events related to endoscopic tattooing.

**Discussion:**

This trial will investigate whether autologous blood marker achieves similar localization accuracy and safety in laparoscopic colorectal surgery compared to intraoperative colonoscopy. If our research hypothesis is statistically proved, the rational introduction of autologous blood tattooing in preoperative colonoscopy can help improve identification of the location of tumors for laparoscopic colorectal cancer surgery, performing an optimal resection, and minimizing unnecessary resections of normal tissues, thereby improving the patient’s quality of life. Our research data will also provide high quality clinical evidence and data support for the conduction of multicenter phase III clinical trials.

**Trial registration:**

This study is registered with ClinicalTrials.gov, NCT05597384. Registered 28 October 2022

## Background

Colorectal cancer (CRC) is the third most common cancer and second leading cause of cancer-related deaths in the world [1]. Laparoscopic surgery has become the standard for management of CRC with the advantages of less traumatic procedure, but similar oncological outcomes to open surgery [1–5]. Due to the lack of tactile perception (haptic feedback), surgeons may have misjudgments in patients with small or flat early colon cancer, malignant polyps resected by endoscopic mucosal resection or endoscopic submucosal dissection. Therefore, the accurate localization of a tumor before surgery is important, especially in the early stages of cancer, to clarify the extent of surgical resection.

Several methods are currently being proposed and used to identify the location of tumors. These include endoscopic tattooing with India ink, indocyanine green (ICG), preoperative endoscopic metal clipping with detection using an x-ray or palpation during surgery [6–14]. Intraoperative endoscopy can ensure proper tumor localization but requires longer operation time and additional healthcare resources, such as another physician or an endoscopy unit in the operating room.

Recently, some retrospective studies reported the use of patients’ autologous blood for preoperative colonic localization in CRC with successful detection by laparoscopy [7]. Autologous blood was thought a feasible and safe tattooing agent for preoperative endoscopic localization. Nonetheless, all currently available evidence comes from observational studies that are susceptible to bias. We therefore proposed to conduct this randomized controlled clinical trial (ABILITY) to evaluate the accuracy and safety of autogenous blood marker localization in laparoscopic radical resection for colorectal cancer.

## Methods/design

### Objectives

The primary objective is to compare the localization accuracy of preoperative endoscopic localization with autologous blood versus intraoperative colonoscopy localization for small, serosa-negative lesion which will be resected by laparoscopic colectomy. The secondary objective is adverse events related to endoscopic tattooing.

### Hypothesis

Preoperative endoscopic localization with autologous blood is no inferiority to intraoperative colonoscopy localization in small, serosa-negative lesion which will be performed by laparoscopic colectomy.

### Study design and setting, and participant

**Fig. 1:**
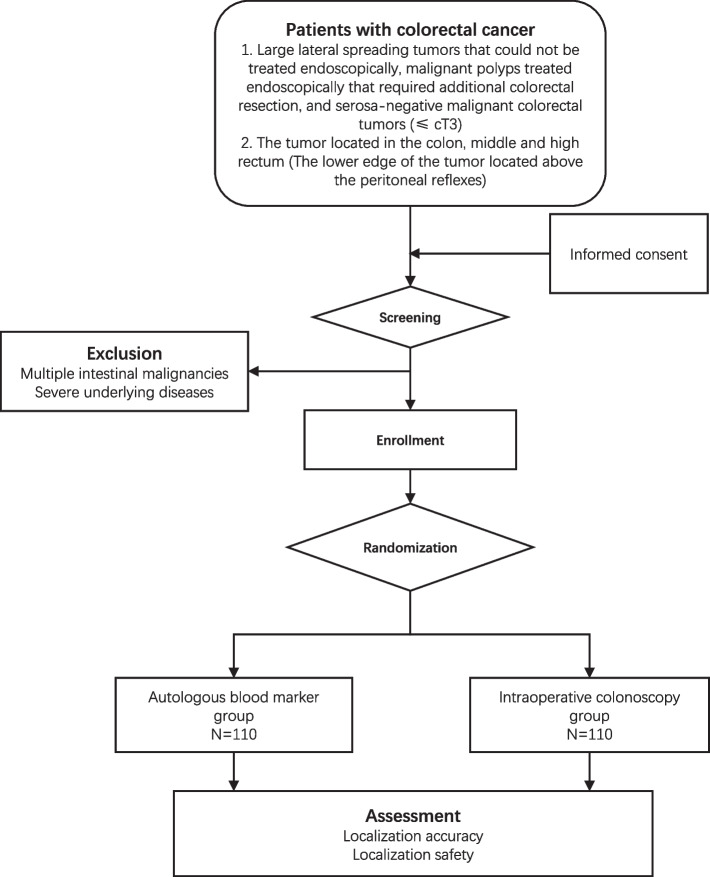
CONSORT diagram

The trial is designed as a randomized, controlled, single center no inferiority trial with two parallel groups and a primary endpoint of localization accuracy during laparoscopic colectomy, which will take place in the Department of Gastrointestinal Surgery, Shanghai East Hospital, Tongji University, Shanghai, China (Fig. 1). The participants will be screened for inclusion and exclusion criteria (Table 1) by the reception oncologist. Eligible patients will be invited for study participation at their first visit at Department of Gastrointestinal Surgery. The doctor who sees the patient will give a formal and detailed description of the study and its procedures. Upon the acquisition of patient written informed consent form, patients will undergo assessment.

**Table 1:** Key inclusion and exclusion criteria of the trial

Inclusion criteria	Exclusion criteria
• Age from 18 to 80 years • Large lateral spreading tumors that could not be treated endoscopically, malignant polyps treated endoscopically that required additional colorectal resection, and serosa-negative malignant colorectal tumors (≤ cT3) • The tumor located in the colon, middle and high rectum (The lower edge of the tumor located above the peritoneal reflexes) • No distant metastasis • American Society of Anesthesiology score (ASA) class I-III • Performance status of 0 or 1 on Eastern Cooperative Oncology Group scale (ECOG) • Written informed consent	• BMI > 35 kg/m2 • Previous history of gastrointestinal surgery that altered the gastrointestinal anatomy. • Pregnant or lactating women • Severe mental disorder • History of previous abdominal surgery (except cholecystectomy and appendectomy) • Rejection of laparoscopic resection • History of cerebrovascular accident within the past six months • History of unstable angina or myocardial infarction within the past six months • History of previous neoadjuvant chemotherapy or radiotherapy • Comorbidity of emergent conditions like obstruction, bleeding or perforation. • Needing simultaneous surgery for other diseases.

After completing an initial assessment and signing an informed consent form, participants will be randomized with 1:1 ratio into two groups: autologous blood marker group and intraoperative colonoscopy group. The data manager, who will not be involved in eligibility assessment and recruitment of patients, will perform the randomization with a list of randomly ordered treatment identifiers generated by a permuted block design using SAS (version 9.1; SAS Institute Inc.). Although it will not be feasible to blind the surgeons and participants, the researcher performing the statistical analyses will be blinded to the patient group allocation.

This protocol and the informed consent forms have been reviewed and approved by Shanghai East Hospital and Institute Ethics Review Committee. We will obtain a new approval from the Committee if any amendments are made to the protocol or the informed consent form that may have an impact on the conduct of the study or potential benefit of the patient. A signed consent must be obtained from every participant in the ancillary study, if the data collection/request is not covered in the original informed consent process for the main clinical trial. The study has been registered in Clinical-Trial.gov (NCT05597384).

### Intervention

All patients will receive localization and surgery within 1 week after randomization. Endoscopic tattooing with autologous blood and intraoperative colonoscopy will be performed by two experienced endoscopists who has more than thousands of cases colonoscopies and more than 200 cases of endoscopic mucosal resection or endoscopic submucosal dissection.

For patients who will enroll in autologous blood group, the tattooing will be performed at 24–48 hours before the surgery. When the lesion is identified by endoscopy, the patient’s peripheral venous blood will be collected using a 10 ml simple syringe without heparin preparation. Immediately after blood sampling, 2–3 ml of autologous blood will be injected submucosally at the distal side and proximal side of the lesion (about 2 cm below and above the border of the lesion) using a conventional endoscopic needle without submucosal injection of normal saline. The tattooing with autologous blood will consider to be invisible if both distal and proximal spots was not identified. For those receiving autologous blood localization, the case will be applied intraoperative colonoscopy if the autologous blood tattoo will not be identified or inaccurate in the laparoscopic colectomy.

For patients who will enroll in intraoperative colonoscopy group, the patient will be placed in the modified lithotomy position under general anesthesia with endotracheal intubation. The legs will be opened and positioned in padded stirrups to facilitate the insertion and manipulation of the colonoscope during the operation. After routine laparoscopic exploration, CO_2_-insufflated intraoperative colonoscopy will be performed using a flexible videocolonoscope. Upstream small bowel clamping will be applied before intraoperative colonoscopy. During intraoperative colonoscopy, CO_2_ pneumoperitoneum will be maintained by the insufflator so that the laparoscope could guide the colonoscope effectively.

After lesion will be identified, a standard laparoscopic colectomy will be performed by two experienced surgeons who has more than 20 years of experience in colorectal surgery with more than 200 cases per year for all enrolled patients. All abdominal operation of laparoscopy will be videotaped. Anastomosis will be performed using the instrumental method. The specimen will be pulled out through a small median incision under the xiphoid (about 3–8 cm).

For those receiving laparoscopic colectomy, the case will be required to be converted to open surgery if one of the following happens: severe or life-threatening intraoperative complications such as intra-abdominal massive haemorrhage, severe organ damage, or other technical or instrumental factors that require a conversion to open surgery.

### Assessment of outcomes

#### Primary outcome

The primary outcome is the localization accuracy. While checking the intraperitoneal cavity at the start of the surgery, the visibility of tattooing will be first checked. After the complete resection of the colon segment, resected colon specimen will be checked the localization with autologous blood tattooing. An accurate localization is defined as tumors identified both by visible tattoo during surgery and by pathological examination of the resected specimen.

#### Secondary outcomes

The secondary endpoint, which is adverse events related to endoscopic tattooing, such as perforation, abscess formation, peritonitis, post-tattoo fever, post-tattoo abdominal pain, and intraperitoneal spillage of tattooing agent, will be evaluated in autologous blood group. Surgery related complications include peritoneal effusion or abscess formation, hemorrhage (inside abdominal cavity, inside digestive tract), ileus, anastomotic leakage, intestinal fistula, lymphatic leakage, gastroparesis, pancreatitis, lung infection, pleural effusion, urinary tract infection, renal failure, liver failure, cardio-cerebrovascular events (both lower extremities thrombosis, pulmonary embolism, myocardial infarction, arrhythmia, cerebral infarction, etc.), and others. Surgical complications will be evaluated in both groups. Complications will be reported and graded according to the Clavien-Dindo classification of surgical complications.

### Data collection

Table 2 displays the schematic diagram for the timeline of patient assessment and data collection. Assessment on localization accuracy and safety will be performed by research physicians and recorded by the research nurse within 30-days after the laparoscopic colectomy. All information should be recorded in Case Report Form (CRF) in a timely, truthful and detailed manner. Researchers must input information into the CRF according to protocol requirements, and supervisors appointed by the research center will check the completeness and accuracy of the CRF and guide the research center personnel to make necessary modifications or additions. CRF will hand over data entry to a reliable medical data processor. Text items can only be checked manually after they are entered into the database from CRF. The data manager performs system checks on the information entered the database. Data will be locked upon completion of entry. Any subsequent changes to the database may only be made with the joint written consent of the clinical study leader, the study statistician, and the data manager.

**Table 2:** Schematic diagram for the schedule of enrolment, interventions, and assessments

TIMEPOINT	STUDY PERIOD			
	Enrolment	Allocation	Post-allocation	
	−1 to −2 weeks	0	1 week	2 weeks
ENROLMENT:				
Eligibility screen	X			
Informed consent	X			
Allocation		X		
INTERVENTIONS:				
*Autologous blood*			X	
*Intraoperative colonoscopy*			X	
ASSESSMENTS:				
*Physical examination*	X		X	
*Laboratory tests*	X		X	
*Oncology assessment*	X			
*localization accuracy*			X	
*Surgical safety*				X

### Sample size and statistical analysis

The primary objective of this study is to identify non-inferiority of autologous blood tattooing versus intraoperative colonoscopy group. Based on our current experience and previous studies which reported the rates of successful visualization, success rates of 99 and 95% were assumed for the intraoperative colonoscopy group and the autologous blood group, respectively. With settings of 80% power, 5% significance level, and non-inferiority margin of 10%, sample sizes of 110 patients in each group will be required.

Continuous variable will be presented as mean and SD if normally distributed, or as median and range otherwise. The differences between the groups were assessed using the t-test, the Mann-Whitney test, Fisher’s exact test, or the χ2 test, as appropriate. Multivariate analysis to assess the impact of potential con- founding factors (sex, age, body mass index, endoscopist, tumor location [colon vs. rectum], clinical tumor stage [≥ T2 vs. T1 or scars]) on invisible tattoo will be performed by using a logistic regression model. All tests will be two-sided with a significance level of *P* < 0.05. All statistical analyses will be carried out using SPSS software package (version 22.0; SPSS Inc.).

### Data monitoring, auditing, and interim analysis

Data monitoring and auditing will be conducted by the funding agency annually. An interim analysis will be performed by an independent statistician when half of the patients have been randomized. The trial will be stopped if one treatment is found to be statistically more beneficial or harmful than the other.

### Adverse events

Adverse events are any unfavourable or unintended events that affect patients on study, regardless of the relevance to the treatment. Any adverse events will be recorded in detail on the CRF regarding its occurrence time, duration, relevance to the treatment, stopping or continuing of the treatment, etc. Events are defined as serious adverse events if leading to death, prolongation of hospitalization, permanent or severe disability, teratogenesis or carcinagensis, and significant clinical sequela [15]. The occurrence of serious adverse events will be reported to Shanghai East Hospital and Institute Ethics Review Committee within 24 h of the initial discovery.

## Discussion

In recent years, early detection rate of early colorectal cancer is gradually increasing. Localization of such small, serosa-negative lesion is an important issue particularly when the surgery is performed through laparoscopy typically compromises tactile feedback [11].

Colonoscopy, endoscopic tattooing with clip or staining agent, and intraoperative colonoscopy can be applied in the appropriate clinical situation to localize colonic lesions. Measurement of the distance from the anus by colonoscopic distancing alone has proven to be an inadequate option in most cases because of colonic redundancy which may cause looping of the scope and increase the distance [9]. Metal clipping combined with abdominal radiography has a limitation in that the metal clips can be lost before the surgery.

India ink is the most widely used material for preoperative tattooing and can persist in tissues in a long time [16], but it may cause inflammatory reactions like peritonitis due to its foreign composition of ethylene glycol, phenols, and animal-derived gelatin [15]. It was reported that the complications resulted from India ink tattooing vary from 0.22 to 14.3% including tattooing agent spillage [17–19]. Intraperitoneal spillage of tattooing agent is not uncommon and can cause the staining of the operation field. Even if no serious complications are observed, it is difficult to collect all the scattered ink in the abdominal cavity. Consequently, foreign material is left permanently in the body [20]. ICG has been used for a long time in tests of cardiac and hepatic function [21], and more recently for sentinel node detection in cancer surgery [22]. ICG is reported an alternative candidate to India ink [23]. It is relatively safer than India ink as a colon tattooing agent [23]. In cases of intraperitoneal spillage, the surgical dissection planes are potentially less compromised under white light [24]. However, ICG tattooing will add significant cost because the imaging requires a special light and camera. Another issue was reported that ICG tattooing maybe ineffective. It was well observed intraoperatively in 95% of the patients within 2 days but tends to disappear within 3 or more days after injection [23]. It was also reported that the high diffusion in the submucosal space was up to 7 cm [25], which could limit the accuracy of tumor localization, and could be expected to fall off relatively early. Other tattooing agents, including methylene blue, indigocarmine, toluidine blue, and isosulfan blue, were visible within a short time after marking, which limit the clinical application. Recently, fluorescent clips, based on traditional endoclips, potentially increases accuracy of localization. Preoperative localization with fluorescent clips could be identified from the serosal side using near- infrared (NIR) cameras. However, the fluorescent clips are rather expensive, but could be expected to fall off relatively early like traditional endoclips [24].

Intraoperative colonoscopy is another precise method; however, it is a somewhat complex procedure that requires an experienced endoscopist and specific instruments in the operating room. Pneumoperitoneum during laparoscopy can reduce visibility of colonoscopy and bowel distension during localization may impair the operative field for the surgeon [26].

Autologous blood was first reported by Korean researchers in 2014 [11]. The effective rates were reported from 90.9 to 92.2% based on several retrospective studies [7, 11, 27]. Autologous blood has advantages of high safety, less inflammation in that it is not a foreign material. No serious tattooing-related complication (such as perforation, colonic abscess formation, or post-tattooing fever) was reported in retrospective studies by now [7, 11, 27]. Complication related to autologous blood was also not found in in our center (data was not shown). Furthermore, this technique is simple, practical, and economic and it does not require a specialized tattooing agent or equipment to detect the target lesion. Mechanical bowel preparation before elective colorectal surgery has been used in our center. To relieve patient discomfort, doing bowel preparation once for both endoscopic tattooing and surgery. Patients receiving autologous blood tattooing underwent surgery within 24–48 h in our center. Some study reported that the longest interval between endoscopic blood tattooing and surgery was 5 days [7]. Another reason that we won’t try longer interval is that many of the previously studied localization agents (including methylene blue, ICG, and others) are absorbed and disappear within 48 h. Autologous blood maybe a good candidate agent for its persistence. We think tattooing with autologous blood can be used worldwide irrespective of the availability of tattooing agents or specialized detection equipment.

The autologous blood tattooing may have disadvantages. The autologous venous blood for tattooing was sampled after identification of the lesion by endoscopy and not prepared to prevent coagulation. If the interval between sampling and injecting is too long, the blood may coagulate. Like other tattooing techniques, intraperitoneal spillage with autologous blood may create difficulty in identifying the tumor and may distort the anatomical tissue planes making laparoscopic dissection more difficult. It was reported that intraperitoneal spillage of tattooing blood results in less staining of the operation field [7]. However, The authors also stated that it was unable to assess the statistical superiority of autologous blood due to the limited number of patients. Data regarding endoscopic tattooing with autologous blood for colorectal surgery are limited. All the studies were retrospective with small sample sizes. Therefore, we proposed this prospective, parallel, two-arm, randomized controlled trial.

India ink is the most widely used material for preoperative tattooing worldwide. We understand trial without India ink is one limitation. Because commercially available autoclave sterilized India ink has not been approved by our hospital right now, we could not conduct a randomized controlled study with India ink. We will compare the autologous blood tattooing with intraoperative endoscopy, which can be candidate method if invisibility after tattooing with India ink in previous studies and ensure proper tumor localization. We also want to perform a trial with India ink with the approve for using the agent in our hospital or cooperate with other hospitals to carry out multi-center research in the future. The current proposed randomized trial therefore aims to evaluate the accuracy and safety of autogenous blood marker localization in laparoscopic radical resection for colorectal cancer. If our research hypothesis is statistically proved, the rational introduction of autologous blood tattooing in preoperative colonoscopy can help improve identification of the location of tumors for laparoscopic colorectal cancer surgery, performing an optimal resection, and minimizing unnecessary resections of normal tissues, thereby improving the patient’s quality of life. Our research data will also provide high quality clinical evidence and data support for the conduction of multicenter phase III clinical trials.

## Data Availability

All study-related information will be stored securely at the study site. The principle investigators had full access to all study data and had final responsibility for the decision to submit for publication. The datasets used and/or analysed during the current study are available from the corresponding author on reasonable request. Participants’ study information will not be released outside of the study without the written permission of the participant. The deidentified datasets generated during the current study will be publicly available via an appropriate data archive 6 months after the completion of the trial.

## Appendix

### Other supplementary information

#### Protocol Version

Issue Date: June 13, 2022

Protocol Amendment Number: 01

Author(s): Shun Zhang, MD; Xiao-hua Jiang, MD

### Sponsor Contact Information

Trial Sponsor: Shanghai Pudong New Area Health Committee and Shanghai East Hospital.

Sponsor’s Reference: Leading talent training program (No. PWR12021–04), and Clinical research project (DFLC2022027)

Address: No. 150 Jimo Road, Pudong New Area District, Shanghai 200,120, China.

Telephone: + 86–21-38,804,518

### Roles and responsibilities

#### Principal investigator and research physicians

Design and conduct of the trial

Preparation of protocol and revisions

Preparation of case report forms

Preparation of materials for international review, board/independent ethics committee applications Patient enrolment

Performing surgery

Publication of study reports

Members of Trial Management Committee

Annual progress reporting to the funder and ethics committee

Serious adverse events reporting to ethics committee Responsible for trial data file

Budget administration

Data verification

#### Data manager

Randomization

Maintenance of trial IT system and data entry Patient follow-up

Data verification

#### Case manager and research nurse

Data collection and patient follow-up

## Abbreviations

CRC: Colorectal cancer
ICG: Indocyanine green
CRF: Case Report Form

## References

[1] Franks PJ (2006). Short-term costs of conventional vs laparoscopic assisted surgery in patients with colorectal cancer (MRC CLASICC trial). Br J Cancer.

[2] Green BL (2013). Long-term follow-up of the Medical Research Council CLASICC trial of conventional versus laparoscopically assisted resection in colorectal cancer. Br J Surg.

[3] Guillou PJ (2005). Short-term endpoints of conventional versus laparoscopic-assisted surgery in patients with colorectal cancer (MRC CLASICC trial): multicentre, randomised controlled trial. Lancet.

[4] Jayne DG (2010). Five-year follow-up of the Medical Research Council CLASICC trial of laparoscopically assisted versus open surgery for colorectal cancer. Br J Surg.

[5] Park JW (2021). Open versus laparoscopic surgery for mid or low rectal cancer after neoadjuvant chemoradiotherapy (COREAN trial): 10-year follow-up of an open-label, non-inferiority, randomised controlled trial. Lancet Gastroenterol Hepatol.

[6] Lee DW (2021). Promising novel technique for tumor localization in laparoscopic colorectal surgery using Indocyanine Green-coated endoscopic clips. Dis Colon Rectum.

[7] Kim EJ (2019). Autologous blood, a novel agent for preoperative colonic localization: a safety and efficacy comparison study. Surg Endosc.

[8] Acuna SA (2017). Preoperative localization of colorectal cancer: a systematic review and meta-analysis. Surg Endosc.

[9] Cho YB (2007). Tumor localization for laparoscopic colorectal surgery. World J Surg.

[10] Committee AT (2010). Endoscopic tattooing. Gastrointest Endosc.

[11] Lee SH (2014). Preoperative localization of early colorectal Cancer or a malignant polyp by using the Patient's own blood. Ann Coloproctol.

[12] Manigrasso M (2022). Preoperative localization in colonic surgery (PLoCoS study): a multicentric experience on behalf of the Italian Society of Colorectal Surgery (SICCR). Updat Surg.

[13] Nguyen NH (2022). Autologous blood for preoperative colorectal TUMOR'S localization: a Vietnamese preliminary experience. Ann Med Surg (Lond).

[14] Offermans T (2017). Preoperative segmental localization of colorectal carcinoma: CT colonography vs. optical colonoscopy. Eur J Surg Oncol.

[15] Feingold DL (2004). Safety and reliability of tattooing colorectal neoplasms prior to laparoscopic resection. J Gastrointest Surg.

[16] Ponsky JL, King JF (1975). Endoscopic marking of colonic lesions. Gastrointest Endosc.

[17] Fu KI (2001). A new endoscopic tattooing technique for identifying the location of colonic lesions during laparoscopic surgery: a comparison with the conventional technique. Endoscopy.

[18] Nizam R (1996). Colonic tattooing with India ink: benefits, risks, and alternatives. Am J Gastroenterol.

[19] Arteaga-Gonzalez I (2006). The use of preoperative endoscopic tattooing in laparoscopic colorectal cancer surgery for endoscopically advanced tumors: a prospective comparative clinical study. World J Surg.

[20] Miyoshi N (2009). Surgical usefulness of indocyanine green as an alternative to India ink for endoscopic marking. Surg Endosc.

[21] Ishigami Y (1993). Clinical applications of ICG finger monitor in patients with liver disease. J Hepatol.

[22] Chen QY (2020). Safety and efficacy of Indocyanine Green tracer-guided lymph node dissection during laparoscopic radical gastrectomy in patients with gastric Cancer: a randomized clinical trial. JAMA Surg.

[23] Lee SJ (2018). Preoperative tattooing using Indocyanine Green in laparoscopic colorectal surgery. Ann Coloproctol.

[24] Barberio M (2021). Preoperative endoscopic marking of the gastrointestinal tract using fluorescence imaging: submucosal indocyanine green tattooing versus a novel fluorescent over-the-scope clip in a survival experimental study. Surg Endosc.

[25] Ushimaru Y (2019). The feasibility and safety of preoperative fluorescence marking with Indocyanine Green (ICG) in laparoscopic gastrectomy for gastric Cancer. J Gastrointest Surg.

[26] Kim SH (1997). Perioperative tumor localization for laparoscopic colorectal surgery. Surg Endosc.

[27] Yeo UD (2020). The usefulness of preoperative Colonoscopic tattooing with autologous blood for localization in laparoscopic colorectal surgery. J Minim Invasive Surg.

